# Quality control of inclusion bodies in *Escherichia coli*

**DOI:** 10.1186/1475-2859-9-41

**Published:** 2010-05-28

**Authors:** Britta Jürgen, Antje Breitenstein, Vlada Urlacher, Knut Büttner, Hongying Lin, Michael Hecker, Thomas Schweder, Peter Neubauer

**Affiliations:** 1 Pharmaceutical Biotechnology, Institute of Pharmacy, Ernst-Moritz-Arndt-Universität, Friedrich-Ludwig-Jahn-Str. 17, D-17487 Greifswald, Germany; 2 Scanbec GmbH, Weinbergweg 23, D-06120 Halle/Saale, Germany; 3 Institut für Biochemie, Heinrich-Heine-Universität Düsseldorf, Universitätsstr. 1, Bldg. 26.02, D-40225 Düsseldorf, Germany; 4 Institute of Microbiology, Ernst-Moritz-Arndt-Universität, Friedrich-Ludwig-Jahn-Str. 15, D-17487 Greifswald, Germany; 5 Institut für Biochemie und Molekularbiologie I, Universitätsklinikum Hamburg-Eppendorf, Martinistr. 52, D-20246 Hamburg, Germany; 6 Bioprocess Engineering Laboratory, Department of Process and Environmental Engineering and Biocenter Oulu, University of Oulu, FIN-90014 Oulu, Finland; 7 Laboratory of Bioprocess Engineering, Department of Biotechnology, Technische Universität Berlin, Ackerstr. 71-76, ACK-24, D-13355 Berlin, Germany

## Abstract

**Background:**

Bacterial inclusion bodies (IBs) are key intermediates for protein production. Their quality affects the refolding yield and further purification. Recent functional and structural studies have revealed that IBs are not dead-end aggregates but undergo dynamic changes, including aggregation, refunctionalization of the protein and proteolysis. Both, aggregation of the folding intermediates and turnover of IBs are influenced by the cellular situation and a number of well-studied chaperones and proteases are included. IBs mostly contain only minor impurities and are relatively homogenous.

**Results:**

IBs of α-glucosidase of *Saccharomyces cerevisiae *after overproduction in *Escherichia coli *contain a large amount of (at least 12 different) major product fragments, as revealed by two-dimensional polyacrylamide gel electrophoresis (2D PAGE). Matrix-Assisted-Laser-Desorption/Ionization-Time-Of-Flight Mass-Spectrometry (MALDI-ToF MS) identification showed that these fragments contain either the N- or the C-terminus of the protein, therefore indicate that these IBs are at least partially created by proteolytic action. Expression of α-glucosidase in single knockout mutants for the major proteases ClpP, Lon, OmpT and FtsH which are known to be involved in the heat shock like response to production of recombinant proteins or to the degradation of IB proteins, *clpP*, *lon*, *ompT*, and *ftsH *did not influence the fragment pattern or the composition of the IBs. The quality of the IBs was also not influenced by the sampling time, cultivation medium (complex and mineral salt medium), production strategy (shake flask, fed-batch fermentation process), production strength (T5-lac or T7 promoter), strain background (K-12 or BL21), or addition of different protease inhibitors during IB preparation.

**Conclusions:**

α-glucosidase is fragmented before aggregation, but neither by proteolytic action on the IBs by the common major proteases, nor during downstream IB preparation. Different fragments co-aggregate in the process of IB formation together with the full-length product. Other intracellular proteases than ClpP or Lon must be responsible for fragmentation. Reaggregation of protease-stable α-glucosidase fragments during *in situ *disintegration of the existing IBs does not seem to occur.

## Background

Fast and high-level expression of heterologous proteins in bacterial hosts results in about 40% of the cases in aggregation and formation of so called inclusion bodies (IBs) [1]. Aggregation occurs as a competitive reaction to folding and therefore depends on the specific folding behaviour and conditions rather than on general characteristics of a protein such as size, fusion partners, subunit structure and relative hydrophobicity [2]. Mostly the target protein is inactive in the IBs, however by rationally performing mutations which influence the aggregation but keep the activity of the protein it is even possible to design IBs with active protein [3].

Aside from the folding behavior of the protein the probability of aggregation *in vivo *is influenced by the cellular set of chaperones and their ability to interfere with the folding intermediates of the target protein. During fast and strong synthesis of a protein with a comparably low folding rate chaperones may be induced by the so called heat shock like response [4-8], but their delayed and often low level synthesis, limit their amount and efficiency, which is different from the natural heat shock response.

Recent studies suggest that the formation of IBs is likely to arise from specific and selective mechanisms, which in part can be compared to amyloid fibril polymerization. Initially this specificity was illustrated by *in vitro *studies with inocculation of nucleation cores [9]. The results of this study support the hypothesis that protein aggregation starts with a slow nucleation phase, possibly through self-assembly of protein monomers via a nucleation-dependent pathway [10] and than microaggregates form bigger IBs. More and more recent data support the similarity between IBs and amyloids (e.g. [11,12]).

Generally IBs are relatively pure. The recombinant product can reach up to 95% of the embedded polypeptides [13,14]. Proteomic analyses revealed that the recombinant protein is relatively homogenous [8,15-17]. Other components which have been detected are traces of nonproteinous ingredients, such as phospholipids and nucleic acids [17], and a background of cellular proteins which are co-isolated by insufficient cell disruption. Especially some membrane proteins are always detected in higher concentration. These are membrane proteins, such as OmpT, and plasmid encoded proteins which are responsible for the antibiotic resistance, such as the kanamycin resistance protein and β-lactamase [8,15]. Membrane components are probably also contaminants retained by unspecific attachment during the purification process [18]. Only a very few cytoplasmic proteins seem to be a real component of IBs - these being the small heat shock proteins IbpA and IbpB in *E. coli *[4,6,13] as well as in minor amounts the chaperones DnaK and GroEL [8,13].

These chaperones play an important and immediate role in the turnover of IBs [19] in addition to the chaperone ClpB, which however has not been annotated so far in electrophoretic separations of the insoluble protein fraction but is observed in the soluble fraction [8,20].

Only recent studies have revealed that the chaperone components in recombinant IBs affect the quality and the turnover very similar to their function in heat shock based protein aggregates. The small heat shock proteins IbpA and IbpB of *E. coli *are intrinsic holding chaperones. It has been proposed that IbpB avoids the inactivation and aggregation of proteins and facilitates their subsequent refolding by DnaK, whereas IbpA seems to mediate the transfer of IbpB together with the non-correcly folded polypeptides into the insoluble cell fraction. It seems that these IbpA and IbpB keep proteins in a folding competent state (avoid irreversible aggregation) and eventually even contribute to enzymatic activity in IBs (Ibp deletion mutants showed no enzymatic activity in IBs) [21]. Also it was shown that IbpA and IbpB decelerated the disintegration of IBs at higher temperatures (37°C), but not at low temperatures (15°C) [20].

DnaK has been localized by immunostaining and transmission electron microscopy on the surface as well as entrapped in the IBs [22]. The importance of the DnaK system in the resolubilization of IBs was shown by Gonzalez-Montalban et al. [23]. The authors demonstrated that IBs in DnaK mutants are toxic and cause inhibition of cell growth. This observation is in line with recent observations by [24] who strongly suggest that the DnaK chaperone system is required for initial substrate unfolding processes at the aggregates, potentially helping to disentangle the entrapped polypeptides to admit them to the central pore of ClpB [24,25]. Although ClpB is probably the central element in the protein reactivation machinery where it acts as a disintegration and refolding chaperone [25,26], these recent results also suggest a very important function for DnaK directly at the surface of a protein aggregate.

In difference to the function of DnaK, the role of GroEL in or on the aggregate is much less clear. Unexpectedly, the deficiency in GroEL results in very small and numerous IBs and more protein amount in the soluble protein fraction [19]. This fact suggests that GroEL could act also as a positive modulator of protein aggregation.

A topic which has not yet been approached very much is the occurrence of fragments of the recombinant protein in the IBs, which have been observed in many cases (e.g. [8,27-31]. These fragments probably represent stable digestion fragments [28] which could be generated either (i) before aggregation by abortive translation, by proteolysis of the newly synthesized polypeptide [27], or (ii) by proteolytic action on or in the IBs, (iii) by reaggregation of stable fragments which are created during the disintegration process of IBs [30], or (iv) during the downstream preparation process of IBs [32]. In case that the fragments contain C-terminal truncations and result from abortive translation, they may be modified by the cellular SsrA tagging system, as in case of human interleukin polypeptides isolated from recombinant IBs [29].

Proteolytic degradation of the polypeptide in many cases has been attributed to the action of the heat shock related ATP dependent proteases Lon and ClpP and it is generally believed that recombinant proteins are better produced in *E. coli *B strains which are naturally Lon minus [33], such as BL21(DE3) [28], and also are negative for OmpT [34]. If proteolysis of cytoplasmically expressed proteins occurred, it had been mainly related to the ClpP protease activity [35]. The effect of other cytoplasmic proteases (for review see [34]) on recombinant products has not been investigated in detail yet. In contrast proteolysis has been more a concern in connection to extracytoplasmic proteases. Especially OmpT has been described as a protease which degrades recombinant products [36,37]. It also can act during purification and refolding, as it is very stable even under strongly denaturating conditions [32]. Other periplasmic proteases which have been assigned to activity on recombinant proteins are DegP (HtrA) [37-39], Ptr [40], and Prc [38].

The appearance of polypeptide fragments of the target protein do not only influence the yield of IBs, but also may affect the refolding efficiency. Therefore we believe that an understanding on the factors which affect the fragmentation in the IBs is of general interest. Earlier, we demonstrated that the α-glucosidase of *S. cerevisiae*, which accumulates during overexpression in *E. coli *exclusively in form of IBs, is remarkably fragmented [8]. In this actual study we investigated by single and double gene knockout strains whether the fragmentation of the α-glucosidase in *E. coli *is due to proteolytic activity of all of the proteases which have been discussed in connection to IB stability, including the two major cytoplasmic proteases ClpP and Lon and the periplasmic proteases OmpT and FtsH. Interestingly, in difference to the many studies cited above, we see that none of these proteases is connected to the fragmentation of α-glucosidase. Due to the detection of fragments which lack the N-termius but have the C-terminus we also can exclude that the key event is pretermination of translation. These studies indicate that other proteases are involved in quality control of recombinant proteins, and consequently, our knowledge on the importance of proteases during the process of recombinant product formation is still incomplete.

## Results

**Figure 1:**
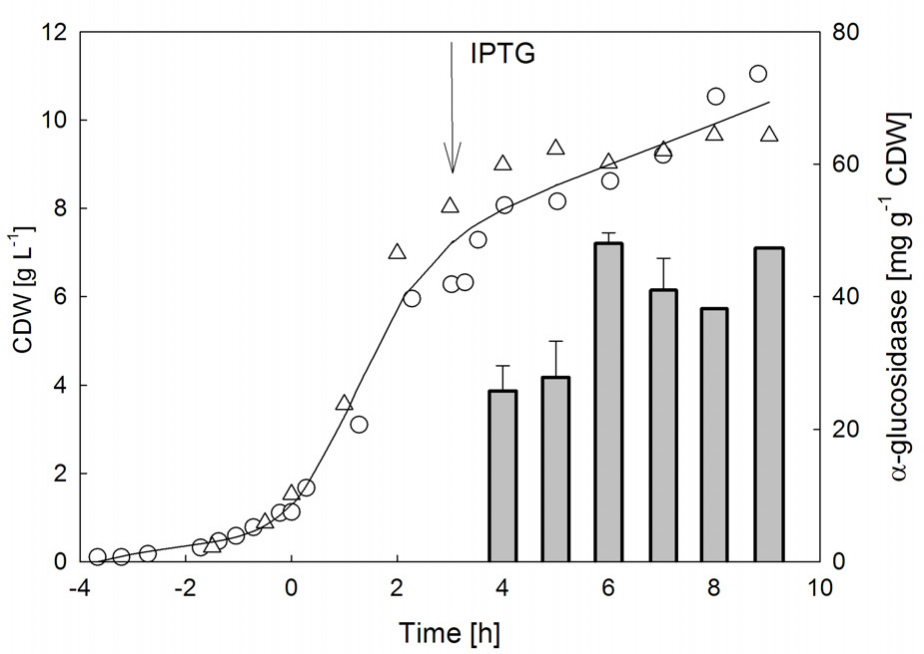
**Profile of a glucose limited fed-batch fermentation with constant glucose feed rate of *E. coli *RB791 overproducing the α-glucosidase after induction of the *tac*-promoter with IPTG**. Data from two different independent cultivations are shown. Cell dry weight (CDW, triangles, squares), α-glucosidase (bars, S.D. of quantified samples).

**Figure 2:**
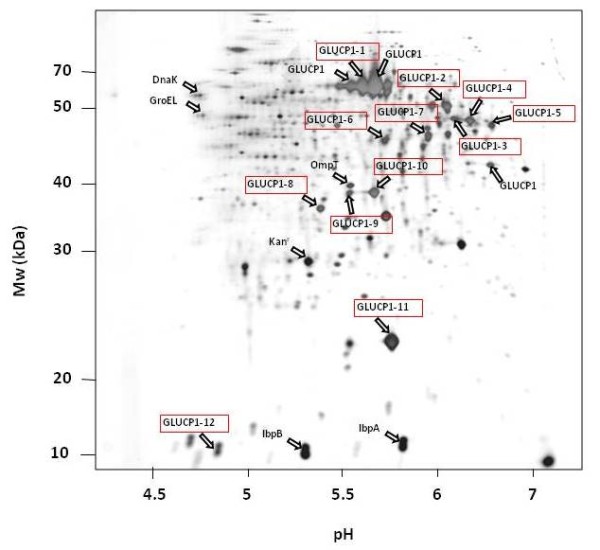
**2D PAGE analysis of the IB protein fraction of the overproduced α-glucosidase (GLUCP1) 3 hours after induction with IPTG**. MALDI-ToF MS analysis and N-terminal sequencing revealed that the majority of the detected protein spot can be assigned to the α-glucosidase (GLUCP1-1 to GLUCP1-12).

Figure 1 shows the profile of a glucose limited fed-batch cultivation of *E. coli *overproducing the α-glucosidase after induction of the promoter with IPTG (figure 1) The α-glucosidase accumulates during overexpression in *E. coli *exclusively in form of IBs (figure 2). No α-glucosidase was found in the cytoplasmic soluble protein fraction (data not shown, see [8]). The aggregated protein fraction yield approximately 20% of total cellular protein.

In order to investigate the composition of the α-glucosidase protein aggregates, the purified IB fraction was separated by means of the 2D PAGE. This analysis revealed that beside the main α-glucosidase protein spot with an expected molecular weight of 68 kDa and an isolelectric point of pH 5.47 a number of additional protein spots could be determined (figure 2).

N-terminal sequencing and the MALDI-ToF MS analyses revealed that most of these protein spots are truncated or modified fragments of the overproduced recombinant α-glucosidase (figure 2). Furthermore, as expected from other studies [4,41], the IB associated proteins IbpA and IbpB were found. Additionally, the kanamycin resistance protein was found to be associated to the α-glucosidase-IBs. Finally, beside the chaperones DnaK and GroEL the outer membrane protease OmpT was found in the aggregated protein fraction (figure 2).

**Figure 3:**
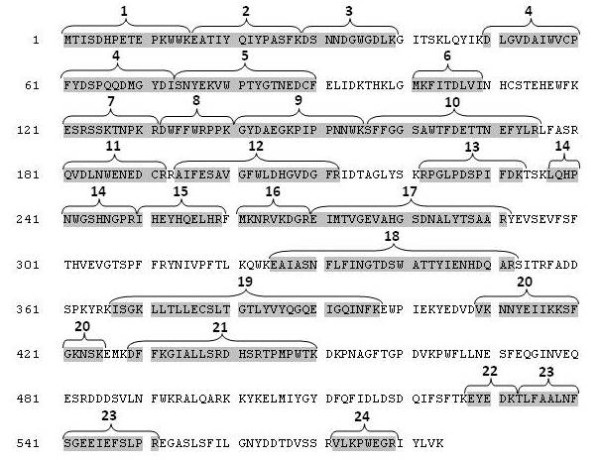
**Sequence coverage of the different peptides of α-glucosidase protein fragments identified by MALDI-ToF MS or N-terminal sequencing (GLUCP1-1 to GLUCP1-12)**. The indentified peptides are serially numbered and marked with brackets which denote the individual fragments of the detected spots (see also Table 1, Additional file 1).

The N-terminal sequencing and the MALDI-ToF MS revealed that at least four of the fragments of the overproduced α-glucosidase lack the N-terminus indicating a proteolytic decay (Table 1, Additional file 1; figures 2 and 3).

No differences in the composition of the IB fraction were observed after growth of the strain in complex medium or in minimal medium or in shake flasks or by fed-batch cultivation in a bioreactor (data not shown). In addition, the sampling time did not affect the characteristic composition of the IB fraction. Samples taken 1 h, 2 h, 3 h or even 4 h after induction of the overproduction of the α-glucosidase exhibit the same protein pattern(data not shown).

**Figure 4:**
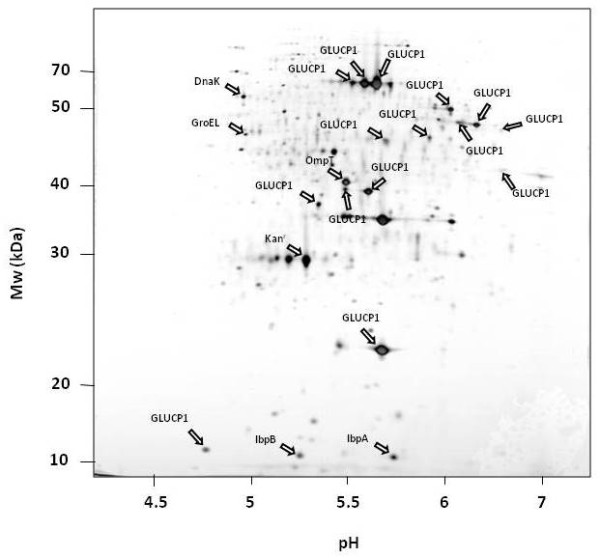
**2D PAGE of the IB fraction of the *E. coli *strain deficient in *rpoS *and *clpP *overproducing the α-glucosidase**.

**Figure 5:**
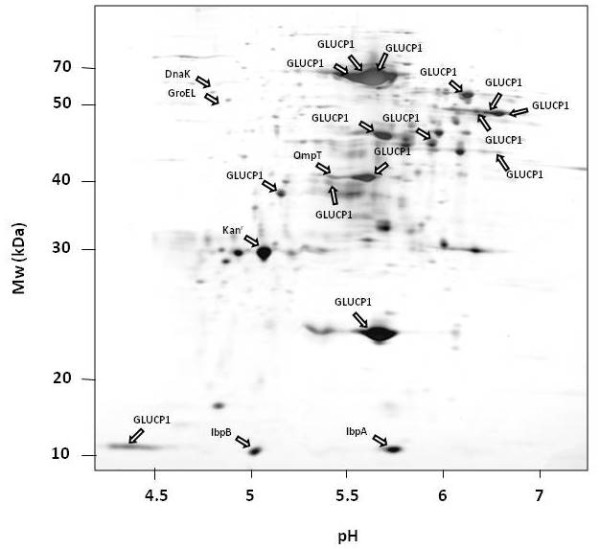
**2D PAGE of the IB fraction of an *E. coli *Lon deficient strain overproducing the α-glucosidase**.

In order to analyze whether the fragmentation of the α-glucosidase could be due to proteolytically degradation by the two major cytoplasmic ATP-dependent proteases of *E. coli*, ClpP or Lon, the IB fraction was investigated in cells lacking these protease genes. Because there is a higher expression of the α-glucosidase in a *clpP/rpoS *double mutant compared to the *clpP *single mutant (own observations, unpublished), the double mutant was used for this analysis. The protein patterns of α-glucosidase IBs derived from *ClpP *(figure 4) and *Lon *(figure 5) deficient strains were very similar compared to the wild type strain-IBs (figure 2). The same α-glucosidase protein fragments as in the control could be determined in both protease mutants.

**Figure 6:**
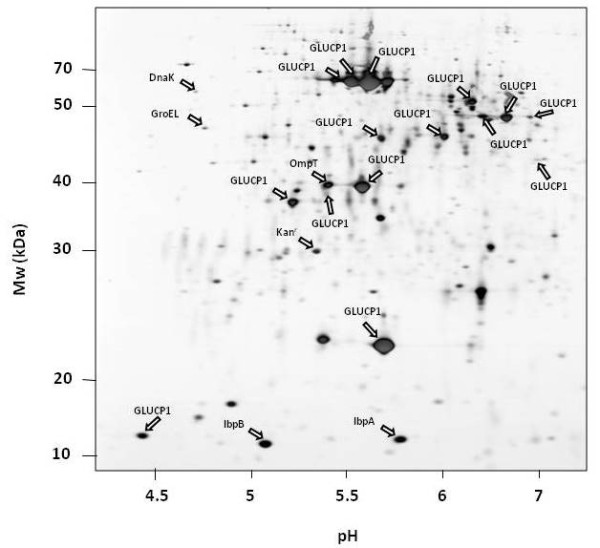
**2D PAGE of the IB fraction of an *E. coli *OmpT deficient strain overproducing the α-glucosidase**.

**Figure 7:**
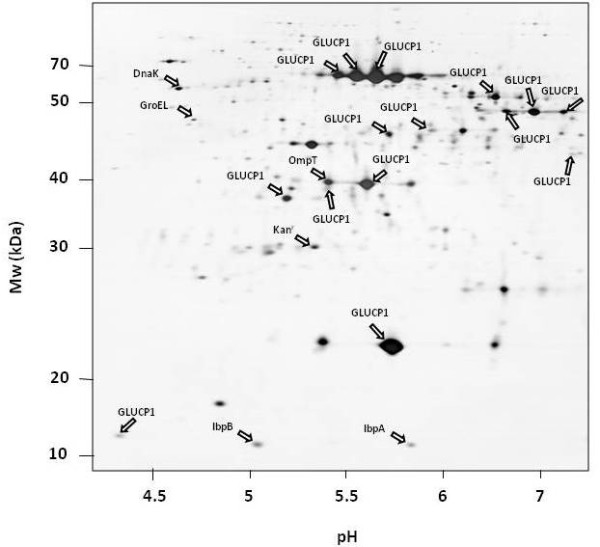
**2D PAGE of the IB fraction of an *E. coli *FtsH deficient strain overproducing the α-glucosidase**.

As shown in figure 2, the protease OmpT was also associated with the IB protein fraction. White et al. [32] demonstrated that OmpT is able to degrade recombinant proteins from IBs during the purification process under extreme denaturing conditions. In order to verify, whether the fragmentation of the α-glucosidase could be due to an artifact during the purification of the insoluble protein fraction, the IBs fraction of an *ompT *mutant was investigated (figure 6). Surprisingly, in comparison to the wild type the α-glycosides protein level in the *ompT *mutant was about fourfold lower (data not shown). However, surprisingly, the fragmentation pattern of the α-glycoside was very similar to that observed in the wild type (figure 2). Finally, the α-glucosidase IB fraction composition of protein samples of an *ftsh *deficient strain was analyzed. Again, the 2D PAGE analysis revealed similar pattern of the IB fraction compared to the wild type strain (figure 7).

Since the major proteases seemed not to have an impact on the IB composition, the effect of different protease inhibitors was tested. For this purpose o-phenantroline, the protease inhibitor cocktail tablets "Complete", EDTA and PMSF were added to the protein samples during IB preparation and 2D PAGE was carried out. O-Phenanthroline and EDTA are known to inhibit metalloproteases [42,43] whereas PMSF block the activity of serine proteases [44]. The inhibition of a multitude of proteases (serine and cysteine proteases) is described for the protease inhibitor cocktail tablets "Complete" (Roche Diagnostics, Germany). In all cases the characteristic composition of the IB fraction with the major α-glucosidase protein fragments was observed (data not shown).

**Figure 8:**
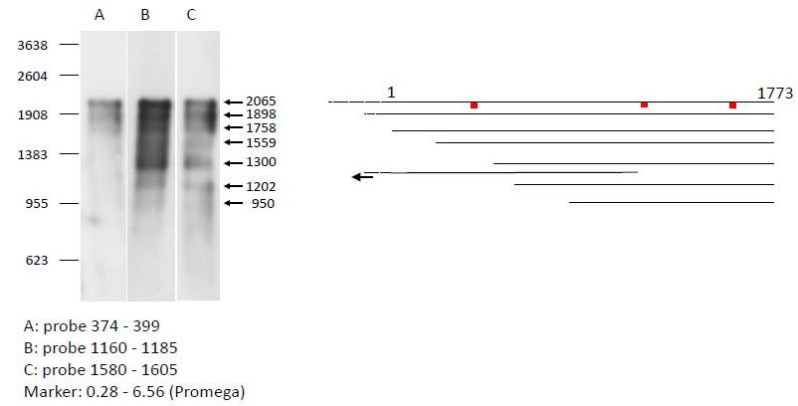
**Northern Blot analyses (A-C) of the α-glucosidase transcript with different oligonucleotide probes**. Analyses were performed with samples from E. coli RB791 pKK177glucC pUBS520 collected from batch cultivations 60 min after induction. Different probes were tested, covering the *glucC *mRNA sequence from position 374 -to 399 (A), 1160 to 1185 (B), and from position 1580 to 1605 (C). The right side of the figure (D) indicates schematically the different locations of the analysed mRNA fragments in relation to the *glucC *sequence. The probe locations are indicated by red bars. Non-translated 5' regions of the mRNA transcript are shown as a dashed line.

Alternatively, as none of the key proteases could be found to be responsible for the fragmentation pattern, we hypothesized that fragmentation products may accumulate due to pretermination of translation. Therefore we performed Northern blot analysis of the *α-glucC *mRNA and analysed the mRNA fragmentation pattern, to investigate whether the α-glucosidase protein fragments with the correct N-terminus might be due to pretermination of translation at non-favorable codons and mRNA degradation at non-ribosome covered regions of the mRNA. Total RNA extracts were analyzed by Northern blot hybridization with three oligonucleotide probes targeting different regions of the *α-glucC *mRNA (positions 374-399, 1160-1185, 1580-1605) (figure 8). The analysis indicated a high fragmentation of the *α-glucC *mRNA with defined fragments of 2065, 1898, 1758, 1559, 1300, 1202, and 950 nucleotides in length. Interestingly, the lowest signals were detected with the 5'-proximal probe although the probe showed similar binding characteristics as the two other probes, indicating a highly unstable 5'-region. As the strongest signals were detected with the probes directed to the central region of the mRNA and to the 5' terminus and as the same RNA fragment patterns were detected for the 3'-proximal probe and the probes situated in the middle of the mRNA or in the 5' region, we conclude that pretermination of translation by stable mRNA fragments is not the reason for the appearance of protein fragments in the IBs.

## Discussion

It is still a common assumption that recombinant proteins, which accumulate in form of IBs, are a homogenous fraction and protected against proteolytic degradation by host cell proteases. Our study demonstrates that this does not hold true in every case. The IB fraction of the insoluble protein investigated in this study, the α-glucosidase of *S. cerevisiae*, was very inhomogeneous. Several host cell proteins like DnaK, GroEL, IbpA, IbpB and OmpT were found to be associated to the protein aggregates. The expression of the small heat-shock proteins IbpA/B is induced during the overexpression of heterologous proteins and both proteins were described as recognizing heterologous protein IBs in *E. coli *cells [4]. Both small heat shock proteins could not be detected in the soluble cytoplasmic protein fraction of α-glycosidase overproducing cells. Rinas and Bailey [15] described the presence of other cellular, non-plasmid-encoded proteins in IB preparations such as the outer membrane proteins OmpF, OmpC, and OmpA or the ribosomal subunit proteins L7/L12. Protein-folding enzymes were not detected in IB preparations. Similar to Rinas and Bailey [45], who found an incorporation of the TEM beta-lactamase precursor into cytoplasmic IBs, we could identify the kanamycin resistance protein in the aggregated protein fraction.

The α-glucosidase seems to aggregate immediately after completion of the translation since no soluble enzyme could be detected in the cytoplasmic protein fraction. The question arose what is responsible for the remarkable fragmentation of the α-glycosidase during the overexpression in *E. coli*? Are there either proteolytic activities associated directly at the ribosomes during translation, allowing a degradation of these proteins already during the translation process, or are there proteases, which are bound to the IBs, allowing the degradation of the aggregated proteins? The later possibility is quite feasible since the cells have evolved mechanisms to remove damaged proteins, for example generated by heat shock or other stresses. For *B. subtilis *it was shown that the major cytoplasmic protease ClpP and their subunits ClpC and ClpX bound to protein aggregates triggered either by heat shock [46] or by overexpression of an insoluble heterologous protein [8]. Ultrathin sections of heat-shocked wild type cells revealed that aggregation damage was significantly decreased or completely disappeared after 30 min of incubation at 50°C, whereas after 30 min *clpC *or *clpP *mutants were as damaged as immediately after the heat stress [46]. In *clpC *or *clpP *mutant cells accumulation of protein aggregates could also be detected under nonstress conditions. These data suggest that in *B. subtilis *ClpP and ClpC play a crucial role in the disaggregation and/or degradation of IBs. The association of chaperones like DnaK and GroEL with the α-glucosidase protein aggregates in *E. coli *indicates that such proteolytic activities directly at the IBs could be possible. These proteins do not only act as chaperones but are also involved in the degradation of proteins, which cannot be folded in a native conformation [47].

Lon and Clp are the major cytoplasmic proteases in *E. coli *[34]. The analysis of the composition of the α-glucosidase IBs in *clpP *and *lon *mutant backgrounds revealed the same fragmentation as in the wild type. This indicates that the two major cytoplasmic ATP-dependent proteases of *E. coli*, Lon and ClpP, are not responsible for the fragmentation of the α-glucosidase in the protein aggregates.

Our analysis demonstrated that the periplasmic protease OmpT was also associated to the α-glucosidase-IBs. It is supposed that the interaction of this protease with the insoluble protein fraction is due to co-purification. Rinas and Bailey [45] found the presence of outer membrane proteins OmpF, OmpC and OmpA in IBs as co-precipitation of cell debris. However, in the case of the α-glycosidase only OmpT but no other outer membrane proteins could be detected in the IB fraction. This could indicate that the presence of OmpT is rather due to a specific activity at the IBs. White et al. [32] described that IB proteins could be degraded by the protease OmpT under extreme denaturating conditions. Our analyses of the IB fraction from an *ompT *mutant demonstrated that the fragmentation of the α-glucosidase is also not due to a proteolytical activity of OmpT.

The *E. coli *FtsH protein is a membrane-bound and ATP-dependent protease. FtsH is involved in the degradation of regulatory proteins such as σ^32 ^and uncomplexed subunits of membrane protein complexes such as SecY of the protein translocase [48,49]. The analysis of the IB fraction of samples of the *ftsH *deficient strain revealed no differences in the protein pattern compared with the wild type strain, indicating that the FtsH protease is not responsible for the fragmentation of the α-glycosidase protein.

Furthermore, rare codons in the sequence of the α-glucosidase could cause the stop of the translation if their appropriate amino acid loaded tRNAs are exhausted. Although the strains used in this study carry plasmid pUBS520, supplying the minor *argU *tRNA at a constant higher level [50], such translational chain breaks during the massive overexpression of the α-glucosidase could be possible. However, as shown by the N-terminal sequencing and MALDI-ToF analyses, there are at least four protein spots lacking the N-terminal part of the α-glucosidase, suggesting that such stops in the translational elongation are not probably. The results of this study underline that IBs of overproduced heterologous proteins are not homogenous fractions of one protein.

## Conclusions

Our data demonstrate that the fragmentation of the α-glucosidase in the IBs is neither due to the major cytoplamic proteases Lon and ClpP nor the detergent stable protease OmpT nor the protease FtsH. Aside from the action of proteases, we also analyzed the probability that protein fragments appear by pretermination of translation caused by fragmentation of the *α-glucC *mRNA. The analysis indicated this to be unlikely, because no specific smaller fragments were detected with probes binding either in the 5'-approximal of the gene or in the middle region in comparison to a probe binding in the 3'-approximal region.

Therefore we suggest that the fragmentation of α-glucosidase is a posttranslational event including other cellular proteases of *E. coli*.

## Materials and methods

### Bacterial strains and plasmids

The *E. coli *strain RB791 [F^-^, IN(*rrnD *- *rrnE*)1, λ^-^, lacI^q^L_8_] was provided from the Genetic Stock Center, Yale University (New Haven). The *E. coli *strains RB791P (RB791, *ΔclpP::cm*, *ΔrpoS::tet*), and RB791L (RB791, *Δlon::tet*) were constructed by P1 transduction of *E. coli *RB791 [51,52]. These strains and strains AR3291 (*ΔftsH3*::*kan*) [48,49] and BL21(DE3) (*lon*^- ^*ompA*^-^) [53] were transformed with plasmid pKK177glucC containing the gene of the α-glucosidase (*glucC*) of *S. cerevisiae *under control of the *tac*-promoter [54] which is induced by addition of isopropyl-β-D-thiogalacto-pyranoside (IPTG). All these strains carry the plasmid pUBS520, supplying the minor *argU *tRNA at a constant higher level and additional copies of *lacI *[50].

### Media and culture conditions

Cultivations were performed in Luria Broth (LB) or phosphate buffered mineral salt medium with glucose (10 g L^-1^) as the only carbon source [55]. The feed solution for the fed-batch cultures contained 200 g kg^-1 ^and the inorganic salts as described by Teich et al. [55]. Additionally 2.75 mL L^-1 ^MgSO_4 _(1 M) was added to the fermenter twice during the feeding phase.

Shake flask cultures were performed in mL Erlenmeyer flasks at 37°C with induction by 1 mM IPTG at optical density (OD)_500 _≈ 0.5. Fed-batch cultures were performed in a 6 L Biostat ED bioreactor (BBI Sartorius, Germany) with an initial culture volume of 4 L. All fermentations were carried out in the mineral salt medium at a temperature of 35°C as described in detail by Teich et al. [55]. The cultivations were started with a batch phase with an initial glucose concentration of 5 g L^-1^. The addition of the glucose feed solution was started shortly before the initially added glucose was exhausted and kept at a constant rate of 53.2 g h^-1^. Induction was performed by addition of 1 mM IPTG 3 h after the start of the feeding. Appropriate antibiotics for initial selection pressure were added to all agar plates, and at the start of all shake flask and fermenter cultivations (appropriate concentrations: ampicillin 100 mg L^-1^, kanamycin 10 mg L^-1^, tetracycline 50 mg L^-1^, chloramphenicol 20 mg L^-1^).

### Two-dimensional polyacrylamide gel electrophoresis (2D PAGE)

Samples were taken two hours after induction of the *tac*-directed expression of the α-glycosidase with IPTG and centrifuged for 10 min at 10,000 rpm. The supernatant was removed and the cell pellet was then washed with 1× Tris-EDTA buffer and stored at -20°C. The proteins were isolated according to the method described by Bernhardt et al. [56]. For analysis of the IBs the cells were disrupted by French Press with 900 PSIG (62.1 bar) followed by centrifugation for 10 min at 14,000 rpm. Then the pellet was washed up to 5 times with IB buffer and resuspended in the same buffer as described by Nurminen et al. [57].

In order to test the impact of protease inhibitors on the preparation of the IB fraction either o-phenanthroline (Merck Chemicals, Germany), ethylenediaminetetra acetic acid (EDTA; 1 mM) (Merck Chemicals, Germany), phenylmethylsulphonyl fluoride (PMSF; 1 mM) (Merck Chemicals, Germany) or the protease inhibitor cocktail tablets "Complete - EDTA-free" (Roche Diagnostics, Germany) were added to the protein samples.

The two-dimensional polyacrylamide gel electrophoresis (2D PAGE) was performed as described in [56]. The protein spots on the gels were identified by means of Matrix-Assisted-Laser-Desorption/Ionization-Time-Of-Flight Mass-Spectrometry (MALDI-ToF MS) with the Voyager DE™ STR of Perspective Biosystems or by N-terminal protein sequencing [58,59] and supported by computer-aided analysis using the software Delta 2D from Decodon (Greifswald, Germany).

### Northern blot analysis

Samples (1 ml cell suspensions) for Northern blot analysis were taken directly from exponentially grown cells 60 min after induction of α-glucosidase synthesis to precooled microfuge tubes containing 0.125 ml inhibitor solution (95:5 ethanol:phenol) to freeze the cell metabolism. After centrifugation for 10 min at 10,000 × g and 4°C total RNA was extracted using the Total RNA kit (A & A Biotechnology, Poland) following the manufacturer's instructions. 1 μg of extracted total RNA was separated on a 1.5% denaturing formaldehyde gel and afterwards blotted onto a positively charged nylon membrane (GE Helthcare, Germany). Northern hybridization was performed overnight at 50°C in High SDS buffer using three oligonucleotide probes (from Metabion, Martinsried, Germany) which were designed to target the α-glucosidase mRNA (*α-gluc *mRNA) (probe 374 - 399: 5'-CCA GUC ACG CUU CGG AUU GGU CUU CCT CCT-3'; probe 1160 - 1185: 5'-AUU GAU CUG GCC UAU CUC CUG ACC UAC TAT-3', probe 1580 - 1605: 5'-AAA CAG CGU CUU GUC UCC GUA CUC UAT CAC-3'). The oligonucleotides (100 pmoles) were labeled with the digoxigenin oligonucleotide 3'- end labeling kit (Roche Diagnostics, Germany) following the standard protocol of the manufacturer. After washing twice with 1 × SSC, 0.1% SDS at room temperature and twice with 0.5 × SSC, 0.1% SDS at hybridization temperature detection was performed by chemiluminescence as recommended by the manufacturer using CDP-Star (Roche Diagnostics, Germany) as substrate and Hyper ECL chemiluminescence films (Kodak, Germany).

## Competing interests

The authors declare that they have no competing interests.

## Authors' contributions

BJ performed the 2D analysis and together with PN the major writing of the manuscript, AB did the Northern Blot experiments, HYL and VU performed the fermentations and sample collection, and KB helped in MS analysis of the protein fragments. PN, TS, and MH supervised the study and participated in its design and coordination and helped to draft the manuscript. All authors read and approved the final manuscript.

## Supplementary Material

Additional file 1 **Table 1**. Overview of the α-glucosidase fragments (GLUCP1-1 to GLUCP1-12) which were detected with the 2D PAGE (see Fig. 2) and their corresponding peptide sequences identified by N-terminal sequencing or MALDI-ToF MS analyses. The localization of the peptides on the α-glucosidase protein sequence is given and the individual peptides are numbered serially. The molecular weight of the particular α-glucosidase protein fragments is estimated from the 2D PAGE or from MALDI-ToF MS analyses. Click here for file
