# Supplementary material for: Quality control of inclusion bodies in Escherichia coli
Source: Microb Cell Fact. 2010 May 28;9:41. doi: 10.1186/1475-2859-9-41 (PMC2893105; doi:10.1186/1475-2859-9-41)
Supplement: Additional file 1 — Table 1. Overview of the α-glucosidase fragments (GLUCP1-1 to GLUCP1-12) which were detected with the 2D PAGE (see Fig. 2) and their corresponding peptide sequences identified by N-terminal sequencing or MALDI-ToF MS analyses. The localization of the peptides on the α-glucosidase protein sequence is given and the individual peptides are numbered serially. The molecular weight of the particular α-glucosidase protein fragments is estimated from the 2D PAGE or from MALDI-ToF MS analyses. [file 1475-2859-9-41-S1.DOC]

Table 1: Overview of the α-glucosidase fragments (GLUCP1-1 to GLUCP1-12) which were detected with the 2D PAGE (see Fig. 2) and their corresponding peptide sequences identified by N-terminal sequencing or MALDI-ToF MS analyses. The localization of the peptides on the α-glucosidase protein sequence is given and the individual peptides are numbered serially. The molecular weight of the particular α-glucosidase protein fragments is estimated from the 2D PAGE or from MALDI-ToF MS analyses.

|  | Fragments ^(A)^ | | | | | | | | | | | | | | | | | | | | | | | | |  |
| --- | --- | --- | --- | --- | --- | --- | --- | --- | --- | --- | --- | --- | --- | --- | --- | --- | --- | --- | --- | --- | --- | --- | --- | --- | --- | --- |
| Fragment  localization | 1 - 15 | 16-28 | 29-39 | 50-73 | 74-90 | 101-109 | 121-131 | 132-140 | 141-155 | 156-175 | 181-192 | 194-212 | 222-233 | 237-249 | 250-259 | 261-269 | 270-291 | 325-352 | 367-397 | 409-425 | 429-450 | 528-532 | 533-551 | 572-579 | Mw [kDa] ^(C)^ | |
| Fragment Nr. | 1 | 2 | 3 | 4 | 5 | 6 | 7 | 8 | 9 | 10 | 11 | 12 | 13 | 14 | 15 | 16 | 17 | 18 | 19 | 20 | 21 | 22 | 23 | 24 | ------- | |
| GLUCP1 -1 |  |  |  |  |  |  |  | x |  | x | x | x |  | x | x |  |  | x |  |  |  |  | x | x | 68.1 | |
| GLUCP1 -2 ^(B)^ |  |  |  |  | x |  | x |  |  | x | x |  |  |  |  | x |  |  | x |  |  | x |  | x | 60.5 | |
| GLUCP1 -3 |  |  |  |  |  |  |  | x |  |  | x | x | x | x | x |  |  | x |  |  |  |  | x | x | 57.3 | |
| GLUCP1 -4 ^(B)^ |  |  |  |  |  | x | x |  |  |  |  |  |  |  |  | x |  |  | x | x | x | x |  | x | 57.3 | |
| GLUCP1 -5 |  |  |  |  |  |  |  | x |  | x |  | x | x | x |  |  |  |  |  |  |  |  |  |  | 52 | |
| GLUCP1 -6 |  | x | x |  |  |  |  | x | x | x | x | x | x | x | x |  | x |  |  |  |  |  |  |  | 45 | |
| GLUCP1 -7 |  |  | x |  |  | x | x | x |  | x |  | x |  | x |  | x | x |  | x |  |  |  |  |  | 47.6 | |
| GLUCP1 -8 | x | x | x |  |  |  | x | x |  |  | x |  | x | x | x |  |  |  |  |  |  |  |  |  | 30.6 | |
| GLUCP1 -9 |  |  |  |  |  |  |  | x |  |  |  |  |  |  |  |  | x |  |  |  |  |  | x | x | 35 | |
| GLUCP1 -10 ^(B)^ |  |  |  |  |  |  |  |  |  |  |  |  |  |  |  | x |  |  | x | x | x | x |  | x | 38.4 | |
| GLUCP1 -11 ^(B)^ |  |  |  |  |  |  |  |  |  |  |  |  |  |  |  |  |  |  |  | x | x | x | x | x | 21.4 | |
| GLUCP1 -12 | x | x | x | x | x |  |  |  |  |  |  |  |  |  |  |  |  |  |  |  |  |  |  |  | 11.633 | |

^(A)^ main fragments; ^(B)^ N-terminal sequencing; ^(C)^ estimated from the 2D gel and/ or from MALDI-ToF analyses
